# Boon of MTA Apexification in Young Permanent Posterior Teeth

**DOI:** 10.1155/2014/673127

**Published:** 2014-10-20

**Authors:** Vinod Kumar, Mohammed Zameer, Vijaya Prasad, T. Mahantesh

**Affiliations:** Department of Pediatric and Preventive Dentistry, Navodaya Dental College and Hospital, Raichur, Karnataka 584103, India

## Abstract

Single visit apexification using mineral trioxide aggregate (MTA) is a new boon in effective management of nonvital tooth with an open apex which has steadily gained popularity with clinicians; also it shortens the treatment period and improves patient compliance. Importance of this approach lies in expedient cleaning and shaping of the root canal system, followed by its apical seal with MTA. There are several case reports available describing the use of MTA as an apexification agent in incisors and premolar, but presented cases are the unique case reports demonstrating successful apexification procedure using MTA in young permanent mandibular molars. After eight-month follow-up, teeth were without any abnormal clinical symptoms; rather there were radiographic resolution of the periapical lesion and induction of root end closure with new hard tissue formation over MTA.

## 1. Introduction

Root development is through the continuous deposition of dentin and cementum by stimulation and differentiation of Hertwig's Epithelial Root Sheath (HERS) and surrounding progenitor cells. Interference in this development by trauma or infection can lead to interruption and arrest of root development which presents with thin and fragile dentinal wall and an absence of natural apical constriction that can create challenging clinical situations. Certainly, cleaning and shaping become difficult and obturation may be unpredictable in the absence of apical stop or a barrier. Therefore, it is essential in these situations to create an artificial apical barrier to allow optimal filling of root canal obturating material avoiding over extrusion. The process is known as apexification. There are several studies [1–4] on the successful use of MTA in one visit apexification treatment on anterior teeth with the outstanding properties of MTA like its biocompatibility, antibacterial property, sealing ability, and potential for regeneration of periradicular tissues [5–15]. Based on these it is considered as an appropriate apexification material [14]. There are very few case reports available in literature of MTA apexification in permanent molars. Thus, the present case reports demonstrate the successful use of MTA to induce root end closure in necrotic young permanent molars.

## 2. Case Reports

### 2.1. Case  1

**Figure 1:**
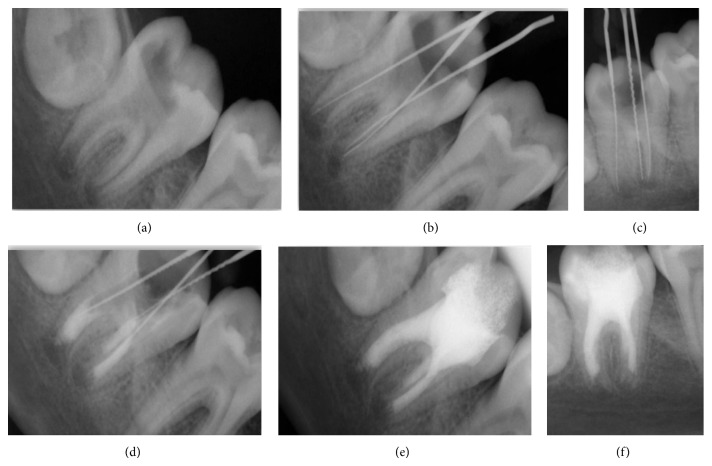
(a) Preoperative. (b) and (c) Working length determination. (d) MTA apical plug of 4-5 mm thickness. (e) Final obturation. (f) After 8-month reassessment, continuous lamina dura and consistent width of periodontal ligament space suggest healing of the periapical lesion.

A 13-year-old boy was reported with pain in the right mandibular 2nd molar 3 weeks ago. Hard tissue examination revealed presence of deep dental caries in relation to the lower 2nd molar. The tooth did not respond to electric and thermal testing. Radiographic examination revealed deep pit communicating with pulp and presence of blunderbuss canals (Figure 1(a)). Apexification using MTA apical plug technique was opted.

Isolation was done using rubber dam and access gained without local anesthesia. Accessible pulpal remnants were removed using barbed broaches. Apex locator produced inconsistent canal length reading so a check radiograph was used to confirm the actual working length (Figures 1(b) and 1(c)). Minimum instrumentation using hand files was done under irrigation using 2.5% NaOCl and saline. Intracanal medicament, that is, Ca(OH)_2_ and iodoform combination dressing, was given. After 2 weeks the patient remained asymptomatic, the tooth was reaccessed, and canals were irrigated. The canals were dried with paper points and MTA was mixed according to the manufacturer's instructions and applied to the apical portion of canal.

The application of MTA was difficult for the posterior teeth due to poor visibility and less accessibility in terms of canal diameter and mouth opening when compared to the anterior teeth. Hence, very small increments were used to enhance precise placement over the apical portion. Initially, 25 gutta-percha cones were used to transfer it to the apical third followed by final condensation using small endodontic plugger. Repeated radiographs were obtained to check adequacy of MTA (Figure 1(d)). The blunt end of a large paper point was moistened with water and left in the canal to promote setting for 4–6 hours. A cotton pellet was placed in chamber and the tooth restored with temporary cement.

After 4–6 hours, the tooth was isolated and accessed as before. A hand plugger was lightly tapped against MTA plug to confirm a hardened set. The canals were dried using sterile paper points and obturated using ZOE sealer and injectable thermoplasticized gutta-percha (Figure 1(e)). Final radiograph was obtained.

After 8-month follow-up, a periapical radiograph was exposed and it revealed that continuity in lamina dura and consistent width of periodontal ligament space suggest healing of the periapical lesion (Figure 1(f)).

### 2.2. Case  2

**Figure 2:**
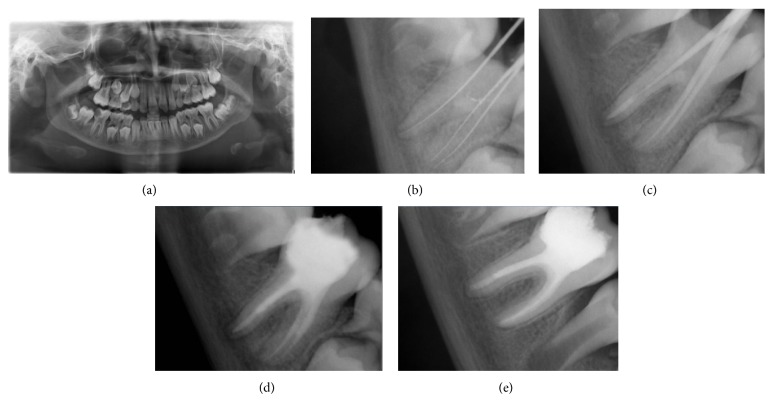
(a) Preoperative. (b) Working length determination. (c) MTA apical plug of 4-5 mm thickness. (d) Final obturation. (e) After 8-month reassessment, continuous lamina dura and consistent width of periodontal ligament space suggest healing of the periapical lesion.

A 9-year-old female patient was reported with pain in the lower right back region 1 week ago. Clinical examination revealed deep dental caries in relation to the right mandibular 1st molar. Radiographic examination revealed deep pit extended to involve pulp with periapical radiolucency and presence of open apex on distal root of the lower right 1st molar (Figure 2(a)).

Tooth isolation, access preparation, extirpation of pulpal remnants, and working length determination were followed the same as case 1 (Figure 2(b)). Complete cleaning and shaping done for the mesial canals but the distal canal were gently debrided by minimum instrumentation under irrigation using 2.5% NaOCl and saline. Application of intercanal medicament and access obtained in case 1 and apexification using MTA were done for only distal root canal (Figure 2(c)). Finally obturation of all 3 canals was done using ZOE sealer and injectable thermoplasticized gutta-percha and final radiograph was obtained (Figure 2(d)).

After 8-month follow-up, a periapical radiograph was exposed for the cases and it revealed that continuity in lamina dura and consistent width of periodontal ligament space suggest healing of the periapical lesion (Figure 2(e)).

## 3. Discussion

Apexification is supposed to create an environment to permit deposition of periodontal tissues to continue root development. However, the conventional apexification material Ca(OH)_2_ has shown inherent disadvantages such as variability in treatment time, unpredictability of apical closure, difficulty in patient follow-up, failure in controlling infection, recurrence of infection, cervical fracture, and increased risk of root fracture [16–18]. MTA has superior biocompatibility and it is less cytotoxic and presence of calcium and phosphate ions results in attraction of blastic cells and promotes favorable environment for cementum deposition [19, 20].

Felippe et al. [21] determined the effect of Ca(OH)_2_ on dogs teeth with open apexes treated with MTA. Their results showed no significant differences in the formation of apical tissue barrier, bone and root resorption, and the presence of microorganisms between the groups. In addition, their findings determined that placing MTA without Ca(OH)_2_ pretreatment results in more complete apical barrier formation compared with those pretreated with Ca(OH)_2_ before placing MTA as an apical barrier. They further demonstrated that the amount of MTA extrusion was significantly higher in samples pretreated with CH compared with those without CH pretreatment. But, there is a well-known “hollow tube” effect in the lexicon of endodontics, where it is thought that an unfilled root canal can be permeated with tissue fluid that becomes stagnant and eventually a nidus for infection [22]. It is stated that Ca(OH)_2_ as a temporary dressing when used between appointments found promotes better results on the periapical healing process that is by efficiently eliminating bacteria which survived after biomechanical instrumentation of the canal [23, 24]. Cwikla et al. in their* in vitro* study determined the antibacterial efficacy of three Ca(OH)_2_ formulations and found Ca(OH)_2_ mixed with iodoform and silicon oil (Metapex) was the most effective dentinal tubule disinfectant [25]. Therefore, in our case reports after minimal root canal preparation Ca(OH)_2_ with iodoform and silicon oil (Metapex) short term dressing was given to disinfect the root canal followed by application of MTA. In contrast to the previous study, our case reports did not show any amount of MTA extrusion.

The microhardness of 2 mm and 5 mm thicknesses of GMTA and WMTA was investigated when the materials were used as an apical barrier. Regardless of the formulation of MTA or placement technique used, a 5 mm thickness was found to be significantly stronger with less leakage than a 2 mm thickness [26]. A scientific article investigated displacement of MTA as an apical barrier material in teeth with open apices, showing that 4 mm thickness of the apical barrier offers significantly more resistance to displacement than 1 mm thickness [27]. This suggests that the thickness of MTA directly affects its hardness, sealing ability, and displacement when used as an apical barrier. Therefore, in accordance with the previous studies, in our case reports 4-5 mm of MTA apical plug was placed.

Aminoshariae and coworkers with radiographic and microscopic evaluation showed that hand method of placement and condensation of MTA resulted in better adaptation with fewer voids than the ultrasonic method [28]. Accordingly, in the present case reports MTA placement and condensation followed manually with pluggers.

Maroto et al. have reported successful apexification with MTA in a tooth that did not respond favorably after 3 years of therapy with Ca(OH)_2_ [16]. In a comparative study on effectiveness of MTA and Ca(OH)_2_ in apexification of traumatized young permanent incisors, MTA demonstrated good success and an effective option for apexification with the advantage of reduced treatment time, good sealing ability, and being biocompatible and provides barrier for immediate obturation [29]. However, MTA is much expensive and more difficult to work with during placement in a root canal due to its naturally sandy consistency when hydrated [30].

## 4. Conclusion

MTA apexification in immature posterior permanent teeth with pulp necrosis and apical pathosis is still difficult in terms of poor visibility, less accessibility due to the narrow canal diameter, and reduced mouth opening when compared to the anterior teeth. However, following the described technique can make it possible to overcome the endodontic challenges in achieving a proper apical plug in posterior teeth.
